# Regenerated isotropic wood

**DOI:** 10.1093/nsr/nwaa230

**Published:** 2020-09-19

**Authors:** Qing-Fang Guan, Zi-Meng Han, Huai-Bin Yang, Zhang-Chi Ling, Shu-Hong Yu

**Affiliations:** Division of Nanomaterials & Chemistry, Hefei National Laboratory for Physical Sciences at the Microscale, Institute of Energy, Hefei Comprehensive National Science Center, CAS Center for Excellence in Nanoscience, Department of Chemistry, Institute of Biomimetic Materials & Chemistry, University of Science and Technology of China, Hefei 230026, China; Division of Nanomaterials & Chemistry, Hefei National Laboratory for Physical Sciences at the Microscale, Institute of Energy, Hefei Comprehensive National Science Center, CAS Center for Excellence in Nanoscience, Department of Chemistry, Institute of Biomimetic Materials & Chemistry, University of Science and Technology of China, Hefei 230026, China; Division of Nanomaterials & Chemistry, Hefei National Laboratory for Physical Sciences at the Microscale, Institute of Energy, Hefei Comprehensive National Science Center, CAS Center for Excellence in Nanoscience, Department of Chemistry, Institute of Biomimetic Materials & Chemistry, University of Science and Technology of China, Hefei 230026, China; Division of Nanomaterials & Chemistry, Hefei National Laboratory for Physical Sciences at the Microscale, Institute of Energy, Hefei Comprehensive National Science Center, CAS Center for Excellence in Nanoscience, Department of Chemistry, Institute of Biomimetic Materials & Chemistry, University of Science and Technology of China, Hefei 230026, China; Division of Nanomaterials & Chemistry, Hefei National Laboratory for Physical Sciences at the Microscale, Institute of Energy, Hefei Comprehensive National Science Center, CAS Center for Excellence in Nanoscience, Department of Chemistry, Institute of Biomimetic Materials & Chemistry, University of Science and Technology of China, Hefei 230026, China

**Keywords:** sustainable structural materials, wood material, cellulose nanofiber, micro/nanoscale structure design, bottom-up strategy

## Abstract

Construction of sustainable high-performance structural materials is a core part of the key global sustainability goal. Many efforts have been made in this field; however, challenges remain in terms of lowering costs by using all-green basic building blocks and improving mechanical properties to meet the demand of practical applications. Here, we report a robust and efficient bottom-up strategy with micro/nanoscale structure design to regenerate an isotropic wood from natural wood particles as a high-performance sustainable structural material. Regenerated isotropic wood (RGI-wood) exceeds the limitations of the anisotropic and inconsistent mechanical properties of natural wood, having isotropic flexural strength of ∼170 MPa and flexural modulus of ∼10 GPa. RGI-wood also shows superior water resistance and fire retardancy properties to natural pine wood. Mass production of large sized RGI-wood and functional RGI-wood nanocomposites can also be achieved.

## Teaser

A new kind of sustainable structural material ‘regenerated isotropic wood’ (RGI-wood) has been prepared from natural wood particles through a bottom-up strategy with surface micro/nanoscale structure design.

## INTRODUCTION

Sustainable high-performance structural materials constructed from all-green (i.e. 100% bio-based) basic building blocks have been attracting more and more interest [1–8]. This research field holds the promise of advancing a key global sustainability goal [1]. However, to date, sustainable structural materials constructed from bio-resources suffer from either limited mechanical properties [1] and dependence on petrochemical-based adhesive (e.g. particle board [9–11], medium density fiberboard [12–14]) or complex manufacturing processes, thus incurring high cost (e.g. polymer-based composites and bio-plastics [1]). Therefore, it is of great importance to introduce an advanced strategy to design and manufacture new kinds of sustainable structural materials.

Micro/nanoscale structure design is an important strategy to improve the properties of structural materials. The large interface area of micro/nanoscale structure and tunable properties of micro/nanoscale interface can facilitate improvement of macroscopic properties of structural materials through this strategy. For example, the toughness of ceramic materials has been improved through the design of a bionic layered structure [15–17], and the fatigue resistance of metal material has been improved through construction of a nanoscale twin structure [18]. Therefore, micro/nanoscale structure design is a powerful strategy with many successful applications [6,19–23], and could play a significant role in designing better sustainable structural materials.

As the most abundant renewable and sustainable resource on Earth, wood has been used for thousands of years for making tools and furniture, and as a construction material. Wood is a natural nanocomposite with aligned cellulose nanofiber embedded in lignin matrix [6], which makes the wood particle an ideal raw material for micro/nanoscale structure design. Here, we employ a robust and efficient strategy with micro/nanoscale structure design to regenerate an isotropic wood from natural wood particles as a high-performance sustainable structural material. This bottom-up process exceeds the limitations of the anisotropic and inconsistent mechanical properties of natural wood, making regenerated isotropic wood (RGI-wood) a strong competitor for engineering plastics. Mass production of large sized RGI-wood can be achieved, overcoming the rarity of large sized natural wood. Using this bottom-up strategy, a series of functional RGI-wood nanocomposites are prepared.

## RESULTS AND DISCUSSION

### Material design and fabrication strategy

**Figure 1.**
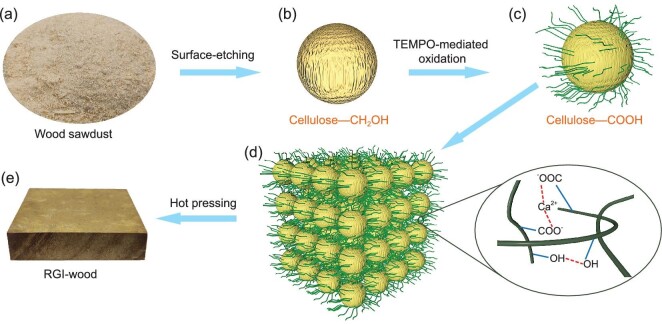
Schematic of the bottom-up approach to regenerate isotropic wood. (a) Natural wood particles, for example, wood sawdust. (b) Surface-etched wood particle with cellulose microfibers exposed from the surface. (c) Surface nanocrystallized wood particle (SNWP) with numerous cellulose nanofibers expanded from the surface. (d) Assembly of SNWP induced by Ca^2+^ and hydrogen bonds. (e) The obtained RGI-wood by hot-pressing.

Figure 1 shows a schematic of our bottom-up approach to regenerate high-performance isotropic wood materials from wood sawdust. The sawdust was first treated in alkali solution to etch the surface to expose the inner cellulose microfiber structure. Then the etched sawdust was washed and went through an oxidation process to convert hydroxyl groups (—OH) of cellulose on the surface into carboxyl groups (—COOH), resulting in disaggregation of cellulose microfiber into nanofibers. Finally, the surface nanocrystallized sawdust was crosslinked with calcium ion and hot-pressed into a high-performance bulk wood material, named ‘RGI-wood’.

**Figure 2.**
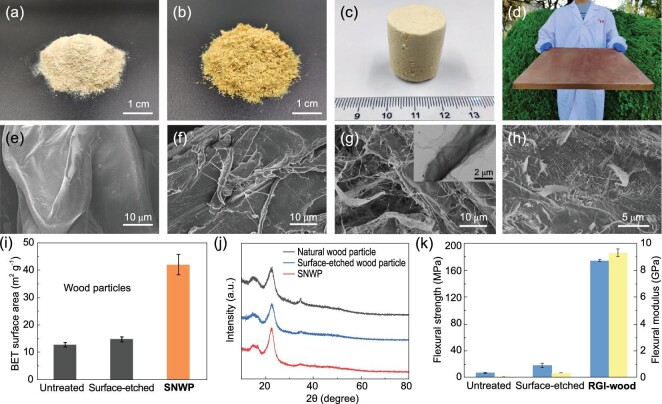
Structure of RGI-wood. Photographs of (a) untreated wood sawdust (natural wood particles), (b) surface-etched freeze-dried wood particles and (c) freeze-dried free-standing sponge of SNWP. (d) RGI-wood sample with a volume of 400 × 400 × 19 mm^3^, weighing 4.1 kg. Scanning electron microscope (SEM) images of (e) untreated wood particles, (f) surface-etched wood particles with the cellulose microfibers exposed from surface, (g) freeze-dried SNWP with numerous cellulose nanofibers expanding from surface and (h) section of RGI-wood sample, a large number of cellulose nanofibers pulling out during the break. (i) The BET surface areas of natural wood particles, surface-etched wood particles and SNWP are 12.63 m^2^ g^−1^, 14.72 m^2^ g^−1^ and 41.99 m^2^ g^−1^, respectively. (j) XRD patterns show that the crystallinity of cellulose of natural wood particles is 63.9 ± 0.70%, whereas that of SNWP is 71.4 ± 0.26%, which confirms a typical feature of cellulose nanofibers. (k) The increasing mechanical performance of different stages of wood particles proved that the outstanding mechanical performance results from the surface nanostructure of the wood particles in RGI-wood. The blue columns represent flexural strength and the yellow columns represent flexural modulus.

In the cell walls of wood, bundles of cellulose nanofibers are combined by hydrogen bonds to form microscale fibers, which are embedded in a matrix of hemicellulose and lignin. Natural wood particles have smooth microscale and nanoscale surfaces (Fig. 2a and e), but cellulose microfibers on the surface can be exposed after partly removing the lignin and hemicellulose from the surface by etching with NaOH and Na_2_SO_3_ solution (Fig. 2b and f). After exposing cellulose microfibers on the surface, the —OH of cellulose are converted into —COOH by TEMPO-mediated oxidation (TEMPO: 2,2,6,6-tetramethylpiperidinyl-1-oxyl), as confirmed by the emergence of a new peak of —COOH in ^13^C-NMR and Fourier transform infrared (FT-IR) spectra (Supplementary Fig. 1). Meanwhile, the swelling of TEMPO-oxidized carboxylated microscale cellulose fibers results in dissociation of the bundle structure and the disaggregation of microscale fibers into nanofibers. Surface nanocrystallization can be achieved by this process.

### Structural characterization of RGI-wood

Surface nanocrystallization is a widely used method to improve the properties of materials, because of the large surface area of nanostructure and the tunable surface physical and chemical properties. After surface nanocrystallization, the morphology and properties of the surface nanocrystallized wood particles (SNWP) change significantly, because of the large surface area of cellulose nanofibers and long-range hydrogen bond interaction between them. It can be observed that many cellulose nanofibers expand from the surface of wood particles (Fig. 2g and Supplementary Fig. 2), indicating successful surface nanocrystallization. The surface area of SNWP dramatically increases to ∼41 m^2^ g^−1^, more than three times larger than that of natural wood particles and surface-etched wood particles (Fig. 2i and Supplementary Fig. 3). X-ray diffraction (XRD) patterns also show that the crystallinity of cellulose in wood particles increases after the surface nanocrystallization (Fig. 2j). The increase in crystallinity is caused by removal of hemicellulose and lignin, which exist in amorphous regions. This results in realignment of cellulose molecules in amorphous regions and increasing crystallinity [24]. The macroscopic properties of SNWP change significantly compared to natural wood particles and surface-etched wood particles as a result of surface nanocrystallization, indicating surface fibrillation of SNWP. The viscosity of SNWP slurry is dramatically increased by at least 2-fold because the cellulose nanofibers on the surface of wood particle form a strong long-range hydrogen bond network (Supplementary Fig. 4). The settling rate of SNWP also slows down visibly for the same reason (Supplementary Fig. 5). Because the surface nanofibers of SNWP can be crosslinked by their abundant hydrogen bonds, the slurry of SNWP can be freeze-dried into a free-standing aerogel directly (Fig. 2c), while only powder is obtained from slurry of natural and surface-etched wood particles (Fig. 2a and b). This slurry of SNWP is further assembled into RGI-wood by hot-pressing in the presence of calcium ion to crosslink the fibers. Through this bottom-up method, large-size RGI-wood can be achieved (Fig. 2d). Unlike any top-down wood processing method, the size of RGI-wood is not restricted by the size of its raw material. In RGI-wood, nanofibers tangle with each other and form hydrogen bonds and ionic bonds on the fiber surface through —OH and —COOH crosslinked by Ca^2+^ (Figs 1d and 2h). Those fibers tightly combine SNWP and make RGI-wood tough and strong. The elimination of pores and the crosslinked nanofiber network results in a relatively higher density of 1.35 g cm^−3^ for RGI-wood than that of natural wood. Surface nanocrystallization greatly enhances mechanical properties of the RGI-wood compared to the bulk of natural and surface-etched wood particles (Fig. 2k).

### Mechanical performance of RGI-wood

**Figure 3.**
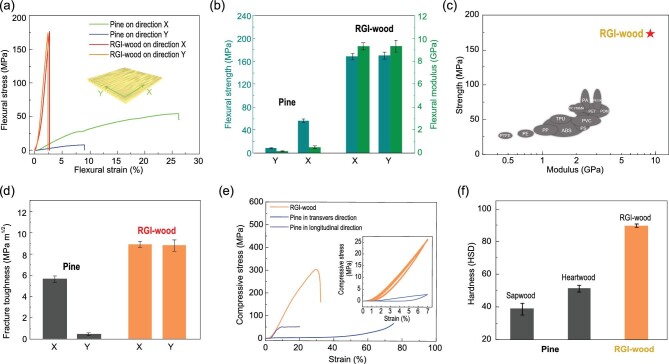
Superior mechanical properties of RGI-wood compared to natural wood. (a) Flexural stress–strain curves of RGI-wood and natural wood in both directions. (b) Compared with natural wood, the RGI-wood has greatly improved strength and modulus. (c) Compared to other polymer-based material, RGI-wood has outstanding mechanical performance, making it a low-cost, high-performance, all-green and environment friendly alternative to plastics. (d) Fracture toughness of RGI-wood and natural wood in two directions. (e) Compressive stress–strain curves of RGI-wood and natural wood. Inset: the stress–strain curves of RGI-wood and natural wood under cyclic compression. The RGI-wood showed neither strength nor modulus change during 10 cycles, whereas the natural wood yields after first compression. (f) Hardness (Shore D hardness) of RGI-wood and different parts of natural wood.

The RGI-wood has a performance superior to that of natural wood such as pine. For pine and other natural wood, mechanical properties are usually anisotropic, as the wood is strong and tough along the growth direction (direction X) but weak and brittle in directions perpendicular to the growth direction (direction Y). RGI-wood has an isotropic uniform structure, which results in identical flexural strength and fracture toughness in both X and Y directions (Fig. 3a and d). As the wood particles are tightly bonded by surface cellulose nanofibers, the flexural strength and modulus can be up to ∼170 MPa and ∼10 GPa, respectively, which is remarkably higher than those of pine in both directions (Fig. 3b). As an all-green biopolymer material, RGI-wood is superior to typical polymers in the main mechanical properties, strength and modulus, making RGI-wood a strong competitor to petroleum-based polymers in many areas of application [25] (Fig. 3c and Supplementary Fig. 6). Despite the lack of long-range orientation of fibers, the fracture toughness of RGI-wood (up to 8.8 MPa m^1/2^) is still higher than that of pine in both directions, because of its strongly bonded nanofiber structure, which can prevent microscale cracks from generation and propagation (Fig. 3d). RGI-wood also has remarkable compression property compared with natural pine wood, which can be attributed to the densification during the bottom-up process (Fig. 3e). The ultimate compressive strength of the RGI-wood is up to ∼300 MPa, and it shows no sign of yielding before being broken. Natural pine wood has a porous structure, which results in yielding under ∼3 MPa during compression. RGI-wood has a compact structure and can endure at least 10 compression cycles under 25 MPa without yielding, which makes it an ideal material for supporting structures in building or furniture. Aside from strength and toughness, the highly crosslinked cellulose nanofibers of RGI-wood also provide a dramatic increase in hardness compared with natural pine wood (Fig. 3f). Sapwood and heartwood of pine have Shore D hardness values of 38.8 ± 3.6 and 51.3 ± 2.5, respectively, whereas that of RGI-wood is up to 89.5 ± 1.0, which also exceeds the values of typical polymers and hardwood and is close to the value of some aluminum alloys (Supplementary Fig. 7). The RGI-wood also provides better impact resistant properties than natural wood, which can be attributed to the highly crosslinked cellulose nanofibers of the RGI-wood and densification during the bottom-up process (Supplementary Fig. 8).

### Superior stability and fire retardancy of RGI-wood

**Figure 4.**
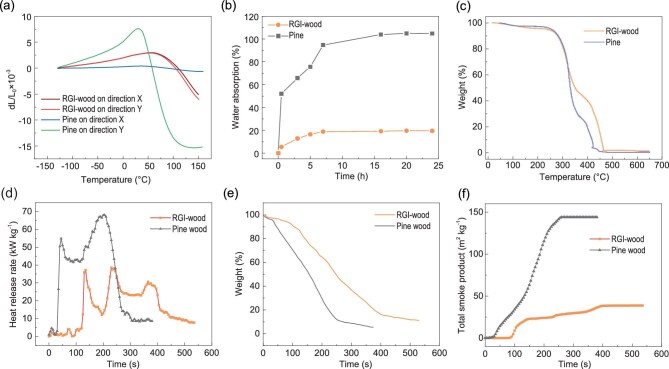
Superior stability and fire retardancy of RGI-wood compared to natural wood. (a) Dimensional stability of RGI-wood and natural wood in both directions under temperature change. (b) The weight gain of RGI-wood and natural wood soaked in water at room temperature for 24 hours. (c) TGA curves of RGI-wood and natural wood show that RGI-wood has higher thermal stability in air. (d) The heat release rate curves of RGI-wood and natural pine wood given by cone calorimetry test showing that RGI-wood releases less heat during combustion. (e) The weight loss curves of RGI-wood and natural pine wood during combustion given by cone calorimetry test showing that RGI-wood loses weight later and slower. (f) The total smoke production curves of RGI-wood and natural pine wood during combustion given by cone calorimetry test showing that RGI-wood has much lower smoke emission.

RGI-wood also has better dimensional stability than natural pine wood under heat or water. Despite the low coefficient of thermal expansion (CTE) of pine wood in the X direction, pine wood shows a high CTE value of ∼48 ppm K^−1^ in the Y direction and dramatically shrinks above 50°C, because of dehydration of bound water (Fig. 4a). The CTE value of RGI-wood is only ∼16 ppm K^−1^, and it begins to shrink at 100°C with a lower shrink rate than pine wood in the Y direction. The improved dimensional stability of the RGI-wood can be attributed to the numerous hydrogen bonds between the nanofibers, which bond wood particles together, and its densified structure. As a uniform polymeric lignin matrix reinforced by cellulose nanofibers, RGI-wood has lower CTE than most typical polymers and aluminum alloys, which is a great advantage for structural material in extremely low or high temperature environments. Water absorption will increase the density and greatly weaken the strength of wood. Because of the porous structure of natural wood, it will absorb a great amount of water in a wet environment, while the densified structure of RGI-wood remarkably reduces its water absorption in a wet environment. Immersion in water at room temperature for 24 hours causes RGI-wood to gain only ∼20% weight, whereas pine wood under the same conditions adsorbs ∼100% weight of water (Fig. 4b).

The inflammability of wood-based materials is another fatal weakness that hinders their use in high-rise building construction [26]. However, the RGI-wood reported here shows superior fire retardancy over natural pine wood, because of its micro/nanoscale structure design. Thermogravimetric analysis (TGA) was employed to evaluate the thermal stability of the RGI-wood and natural pine wood (Fig. 4c and Supplementary Fig. 9a). Both show three main stages during pyrolysis in air, defined as dehydration, oxidative pyrolysis and char oxidation, respectively [27]. RGI-wood loses less weight during oxidative pyrolysis as its compact structure results in a higher yield of char and less volatilization. The higher char yield means that the RGI-wood possesses better fire retardancy than natural wood. Furthermore, cone calorimetry has been used to evaluate the fire retardancy under real-world fire conditions. A series of important parameters of the RGI-wood obtained from cone calorimetry tests are superior over natural pine wood, such as time-to-ignition (TTI), heat release rate (HRR), total heat release (THR), total smoke production (TSP) and weight loss. The ignition time of wood-based materials is a core fire-retardant property. Longer ignition time can effectively slow down the spread of fire in real-world fire ground. Much successful work has been done to increase the ignition time of wood-based materials. For example, Hu *et al*. [28] achieved excellent fire-retardant property of 2.08-fold enhancement in TTI by densification of bulk wood. Compared with natural pine wood, a 4-fold enhancement in the TTI of RGI-wood was achieved. The TTI of the RGI-wood is 120 s under heat flux of 35 kW m^−2^, while natural wood is ignited within 29 s under the same conditions, meaning that it is more difficult to ignite the RGI-wood. The significantly delayed combustion peaks shown in HRR and THR curves suggest enhanced fire safety (Fig. 4d and Supplementary Fig. 9b). After ignition, the RGI-wood has a lower heat release rate, which results in lower heat feedback, lower surface temperature and slower fire spread. Three peaks, instead of two for natural wood, are shown in the HRR curve for RGI-wood, indicating generation of a fireproof carbonized layer and the temporary extinguishing of fire. The RGI-wood loses weight later and slower than does natural wood during combustion, because of its compact nano-network (Fig. 4e). In addition, toxic smoke emission is another major problem of combustion. However, enhanced fire retardancy usually dramatically increases smoke production because of addition of chemical fire retardant and incomplete combustion [29]. With its enhanced fire retardancy, the RGI-wood produces 74% less smoke than natural wood, which can be attributed to its dense structure preventing inside smoldering (Fig. 4f and Supplementary Fig. 9c and d).

It should be noted that this bottom-up strategy with micro/nanoscale structure design to prepare bulk structural materials is totally different from a top-down strategy with chemical treatment to process bulk natural wood [6]. The preparation of compressed wood is a top-down strategy with chemical treatment to process bulk natural wood, which is limited by the anisotropy and size of natural wood. In contrast, our bottom-up strategy with micro/nanoscale structure design exceeds the limitations of anisotropic, inconsistent mechanical properties and sizes of natural wood by introducing a surface nanocrystallization method of different kinds of biomass particles. Compared with a top-down strategy, our bottom-up strategy with micro/nanoscale structure design is more efficient, low cost and can be scaled up to commercial process directly. Furthermore, through this bottom-up processing strategy, a series of functional nanoscale building blocks can be mixed with surface nanocrystallized wood particles before hot-pressing to prepare functionalized RGI-wood nanocomposites in an effective way, whereas is difficult to achieve uniform mixing of functional nanoscale building blocks with compressed wood using a top-down method [6]. Thus, our work is totally different from the work of processing bulk wood into high-performance structural materials.

### Conductive smart RGI-wood

**Figure 5.**
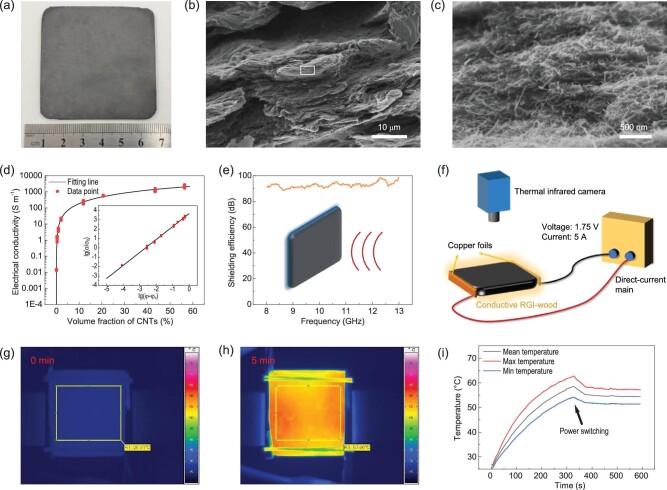
Conductive smart RGI-wood. (a) Photograph of conductive smart RGI-wood. (b) SEM image of conductive smart RGI-wood with dense structure. (c) CNT-covered SNWP in conductive smart RGI-wood (enlarged micrograph of the marked area in (b)). (d) Conductivity of the conductive smart RGI-wood to volume fraction of CNT curve and fitting line. (e) Electromagnetic shielding performance of the conductive smart RGI-wood. (f) Diagram of self-heating device based on conductive RGI-wood. (g) Thermal infrared image of self-heating device before heat. (h) Thermal infrared image of self-heating device after 5 minutes at a voltage of 1.75 V. (i) Temperature of conductive smart RGI-wood at a voltage of 1.75 V and a current of 5 A. After the heating process, switching the voltage to 1.4 V can maintain the sample temperature at 55°C, making it a good smart self-heating board.

Through this bottom-up processing strategy, a series of functionalized RGI-wood nanocomposites can also be prepared by mixing SNWP with functional nanoscale building blocks before hot-pressing. Taking the conductive smart RGI-wood composited with carbon nanotubes (CNTs) as an example, it can be seen from the scanning electron microscope (SEM) images that the CNTs are evenly wrapped around the outer layer of the wood particles (Fig. 5a–c). Because of the extrusion of the wood particles, the CNTs are highly densely packed and form an efficient conductive network in the RGI-wood. The conductivity of this conductive RGI-wood can be described using a power–law relationship and three-dimensional percolation theory as [30]:

(1) \begin{equation*} \sigma = {\sigma _{\it 0}}{(\upsilon - {\upsilon _c})^t}, \end{equation*}

where *σ* is the conductivity of nanocomposite, *σ_0_* is a scaling factor proportional to the intrinsic conductivity of the filler, *υ* is the volume fraction of conductive filler and *υ_c_* is the volumetric fraction at the percolation threshold, and *t* is the critical exponent of the conductivity. The best fit of the conductivity data to the laws of power give a percolation threshold of ∼0.06 vol.% and a critical exponent of ∼1.38 according to Eq. (1) in the conductive RGI-wood (Fig. 5d).

Compared with most CNT/polymer composites [31], the conductive RGI-wood has a lower percolation threshold and a lower exponent, indicating that CNTs can form a better conductive network between the wood particles than evenly distributing in a polymer matrix. A diagrammatic sketch shows that extrusion of the microscale wood particles can reduce remarkably the contact resistance and even distribution of CNTs leads to ‘dead arms’ [32–34], which can reduce the effective concentration of CNTs and result in lower conductivity (Supplementary Fig. 10). Because of its high conductivity, this intelligent conductive RGI-wood shows excellent electromagnetic shielding performance. When the content of CNTs is about 18.5%, the electromagnetic shielding effectiveness of 2 mm thick conductive RGI-wood exceeds 90 dB in the X-band, which meets the requirements of shielding standards of precision electronic instruments (Fig. 5e). This excellent electrical conductivity of intelligent conductive RGI-wood also allows it to self-heat through Joule heat at low voltages. As shown in Fig. 5f, the device consists of a direct-current main and a conductive RGI-wood sample with two copper foils stuck to it by conductive silver paint. A thermal camera is used to measure the thermal field on the device surface during the heat process. A sample of size 60 × 60 × 2 mm^3^ can be heated from room temperature to 60°C in 5 minutes under 1.75 V (Fig. 5i). The thermal image indicated that the device was evenly heated and no hot point or cold point could be observed (Fig. 5g and h). A low heating voltage can effectively ensure the safety of self-heating devices while reducing energy consumption. This device based on the Joule heat and ‘smart’ conductive RGI-wood can be used as self-heating wallboard for smart buildings.

## CONCLUSION

In summary, a bottom-up strategy with micro/nanoscale structure design has been proposed to construct high-performance sustainable structural materials directly from wood particles. The obtained RGI-wood has many remarkable properties, including high flexural strength, high flexural modulus, high toughness, high compressive strength, high hardness, good dimensional stability and fire retardancy (Supplementary Fig. 11). This versatile bottom-up strategy with micro/nanoscale structure design enables efficient mass production of various sustainable structural materials with superior properties to natural wood by formulating the components based on biomass, which exceeds the limitations of the anisotropic and inconsistent mechanical properties and inflammability of natural wood.

## METHODS

### Fabrication of RGI-wood

All reagents and raw materials were commercially available. The sawdust of pine wood (*Pinus sylvestris Linn. var. mongolica Litv.*) was first immersed in an aqueous solution of mixed 2 M NaOH and 0.4 M Na_2_SO_3_ for 5 hours, followed by rinsing one time with deionized water. Second, surface-etched wood particles were surface oxidized by TEMPO-oxidized method [35] (TEMPO: 2,2,6,6-tetramethylpiperidinyl-1-oxyl). Finally, the surface nanocrystallized wood particles (SNWP) were immersed into CaCl_2_ solution and hot-pressed at 95°C with pressure of 100 MPa until RGI-wood was completely dry. By mixing SNWP with different kinds of basic building blocks (e.g. CNTs, MMT, glass bubbles), different kinds of RGI-wood composites can be obtained.

### Characterization

SEM images were taken with a Carl Zeiss Supra 40 field emission scanning electron microscope (5 kV). All samples were gold sputtered with gold for 30 seconds at a constant current of 30 mA before observation. XRD data were measured by a PANalytical X’pert PRO MRD X-ray diffractometer equipped with Cu Kα radiation (λ = 1.54056 Å). The RGI-wood was tested using a concave stage, and the sawdust, surface-etched wood particles and SNWP were ground before test. TGA data were measured in air atmosphere with a TA Instruments SDT Q600 thermogravimetric analyzer. The samples were prepared by grinding natural wood and RGI-wood into powders. FT-IR spectra were acquired by a Bruker Vector-22 FT-IR spectrometer in attenuated total reflectance (ATR) mode. The samples were ground into powders. Nuclear magnetic resonance (NMR) ^13^C CPMAS spectra were recorded at 100.62 MHz on a Bruker Avance III 400 WB spectrometer using a 90° pulse of 4 μs, acquisition time of 33.9 ms, contact time of 3 ms, recycle delay of 5 s and a spin rate of 14 kHz. Three-point bending test was performed on an Instron 5565A universal testing machine according to ASTM D790-15e1. The specimens were carefully cut to a size of about 25 mm × 3 mm × 3 mm. The test was carried out at room temperature with a loading rate of 1.0 mm min^−1^ and a support span of 12.5 mm. Electromagnetic shielding effectiveness was measured at a frequency of 8–13 GHz by TE 10 waveguide techniques. The specimen size was about 60 mm × 60 mm × 3 mm. The fire retardency properties of specimens were investigated on a FTT0007 cone calorimeter according to ISO 5660-1 standard, with a heat flux of 35 kW m^−2^. The dimensions of the samples were 60 mm × 60 mm × 6 mm.

## Supplementary Material

nwaa230_Supplemental_File
