# Supplementary material for: Regenerated isotropic wood
Source: Natl Sci Rev. 2020 Sep 19;8(7):nwaa230. doi: 10.1093/nsr/nwaa230 (PMC8310772; doi:10.1093/nsr/nwaa230)
Supplement: nwaa230_Supplemental_File [file nwaa230_supplemental_file.docx]

Supplementary Information for

# Regenerated isotropic wood

Qing-Fang Guan^1†^, Zi-Meng Han^1†^, Huai-Bin Yang^1†^, Zhang-Chi Ling^1^ & Shu-Hong Yu^1^*

*^1^ Division of Nanomaterials & Chemistry, Hefei National Laboratory for Physical Sciences at the Microscale, Institute of Energy, Hefei Comprehensive National Science Center, CAS Center for Excellence in Nanoscience, Department of Chemistry, Institute of Biomimetic Materials & Chemistry*, *University of Science and Technology of China, Hefei 230026, China.*

*Correspondence to: Shu-Hong Yu ([shyu@ustc.edu.cn](mailto:shyu@ustc.edu.cn)).

† These authors contributed equally to this work.

**This PDF file includes:**

Characterization and testing

Supplementary Figures 1-11

**Characterization and testing**

**Single-edge notched bend test (SENB).**

SENB test was performed on an Instron 5565A universal testing machine. The specimens were firstly cut with the size of about 25 mm × 3 mm × 3 mm. The specimens were notched to approximately half (45-55 %) of their widths using a diamond saw (~150 μm), and then the notch was sharpened by slightly sliding a razor blade repeatedly. The test was carried out at room temperature with a loading rate of 1 μm s^−1^ and a support span of 12.5 mm.

**Hardness test.**

Hardness of specimens was measured by Shore D Durometer.

**Compression test.**

Compression test was performed on an Instron 5565A universal testing machine. The specimens were carefully cut with the size of about 3 mm × 3 mm × 3 mm. The test was carried out at room temperature with a displacement rate of 1 mm min^-1^.

**Drop hammer impact test.**

The drop hammer impact test of RGI-wood was performed on an Instron CEAST 9340 drop hammer tester. The hammer with 15 kg (total mass) weight was falling freely from 30 cm height, and the dimensions of the tested specimens were about 50 mm × 50 mm × 2 mm.

**Viscosity test.**

Viscosity of slurry of three kinds of wood particles with 6 wt. % solid content were measured by Rotational viscometer NDJ-1 at 25 °C.

**Brunauer-Emmett-Teller (BET) analysis.**

BET analysis of wood particles was run on a Quantachrome autosorb iQ-MP/XR and N_2_ desorption at 77.35 K was utilized to determine the specific surface area. From the obtained isotherms the BET surface area was calculated by multi-point BET.

**Calculation of thermal expansion coefficient.**

Thermal expansion coefficients of specimens were measured by TMA Q400. The specimens were carefully cut with the size of about 20 mm × 3 mm × 3 mm and the test was carried out from -130 °C to 150 °C. Thermal expansion coefficient (α) was calculated by the equation *α* = *ΔL* / (*L* *ΔT*) K^-1^.

**Electrical conductivity measurements.**

The electrical conductivity of specimens were measured by a two probe method with a multimeter at room temperature in air. A layer of Ag paste was uniformly pasted on two opposite sides of the samples as electrode pairs.

**Thermal diffusion coefficient.**

Thermal diffusion coefficient is measured by Hot Disk TPS 1500 S. The dimensions of the samples were 60 mm × 60 mm × 6 mm.

**Calculation of the density.**

Density (*ρ*) of the RGI-wood was calculated by first treating the material into a cuboid, and then using the equation *ρ* = mass/volume. The specific values of the impact fracture toughness and the ultimate flexural strength were calculated by dividing the density.

**Calculation of the fracture toughness.**

The fracture toughness can be calculated by SENB test. Fracture toughness for crack initiation (*K*_Ic_) was calculated by

$K_{\mathrm{Ic}}=\frac{P_{\mathrm{Ic}}S}{BW^{3/2}}f\left( a/W \right)$ , (1)

where *P*_Ic_ represents the maximum load in SENB test, *S* the span, *B* the thickness of the SENB specimen, *W* the width and *a* the length of the pre-crack. The function *f* is given by

$f\left( a/W \right)= \frac{3{(a/W)}^{1/2}[1.99-\left( a/W \right)\left( 1-a/W \right)\left( 2.15-3.93a/W+{2.7(a/W)}^{2} \right)]}{2(1+2a/W){(1-a/W)}^{3/2}}$ . (2)

The maximum fracture toughness (*K*_Jc_) is determined by

$K_{Jc}={[(J_{\mathrm{el}}+J_{\mathrm{pl}})E']}^{1/2}$ , (3)

where *J*_el_ is the elastic component of *J*-integral, *J*_pl_ the plastic component of *J*-integral and *E'* is given by

$E^{'}=E(1-v^{2})$ , (4)

where *E* is the elastic modulus and *ν* the Poisson’s ratio.

The elastic component *J*_el_ is based on linear elastic fracture mechanics.

$J_{\mathrm{el}}=\frac{K_{\mathrm{Ic}}^{2}}{E^{'}}$ . (5)

The plastic component *J*_pl_ is calculated by

$J_{\mathrm{pl}}= \frac{2A_{\mathrm{pl}}}{B(W-a)}$ . (6)


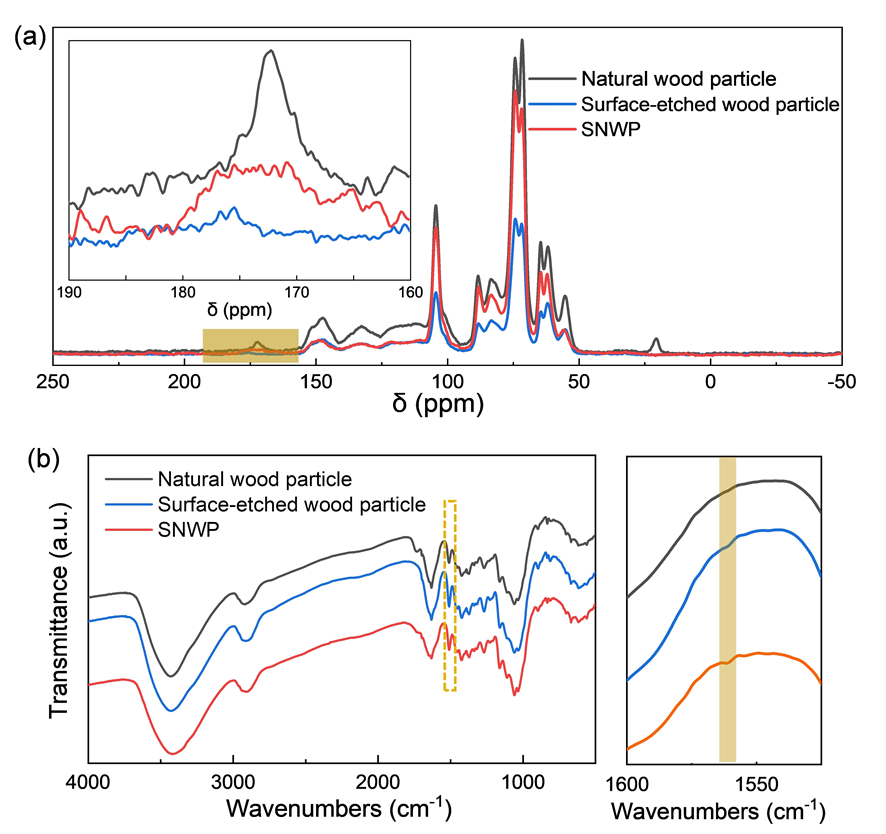


**Supplementary Figure 1. Comparison of ^13^C nuclear magnetic resonance (^13^C-NMR) and Fourier transform infrared spectroscopy (FT-IR).** (**a**) The peak between 170-175 ppm of surface nanocrystallized wood particles (SNWP) can be attributed to carboxyl groups (-COOH) while surface-etched wood particles show no peak in this range, showing that oxidation process converts -OH of cellulose into -COOH on the surface of wood particles^1^. (**b**) FT-IR spectrum shows that new -COOH groups emerge after oxidation.


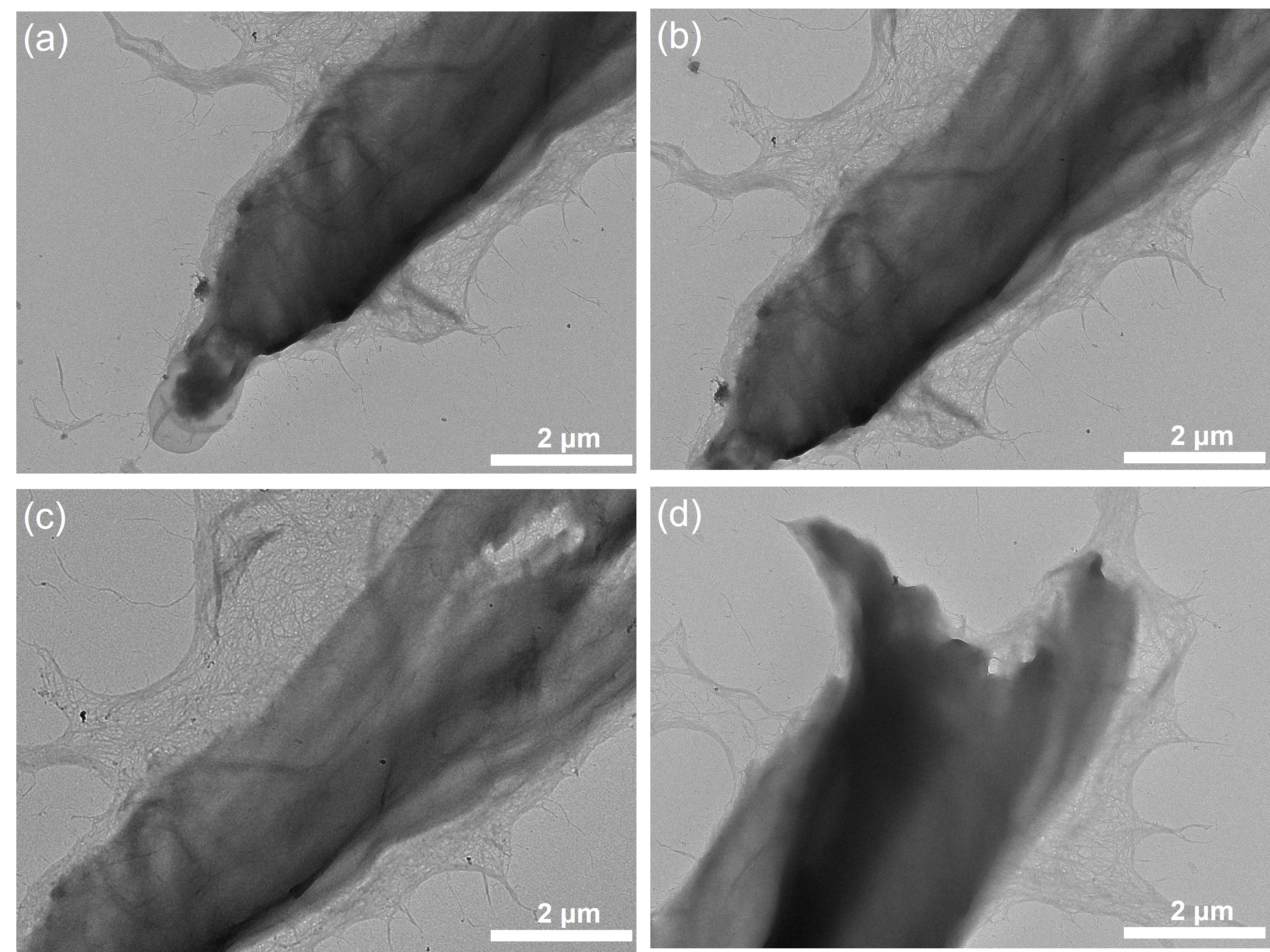


**Supplementary Figure 2. The transmission electron microscope (TEM) images of SNWP.** (**a-d**) The TEM images of SNWP.


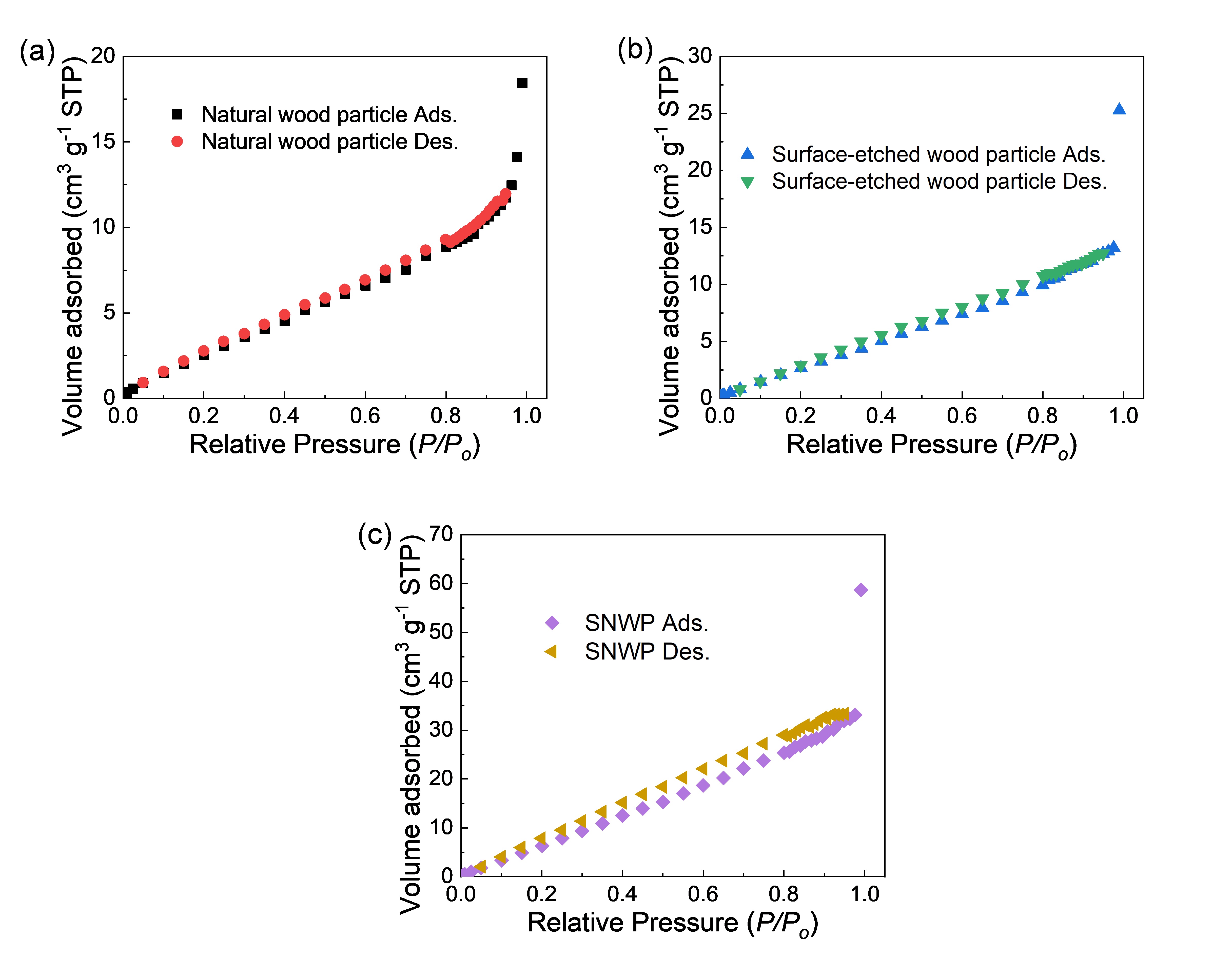


**Supplementary Figure 3. The impact of surface treatment on porosity.** Nitrogen isotherms for (**a**) natural wood particles, (**b**) surface-etched wood particles and (**c**) SNWP.


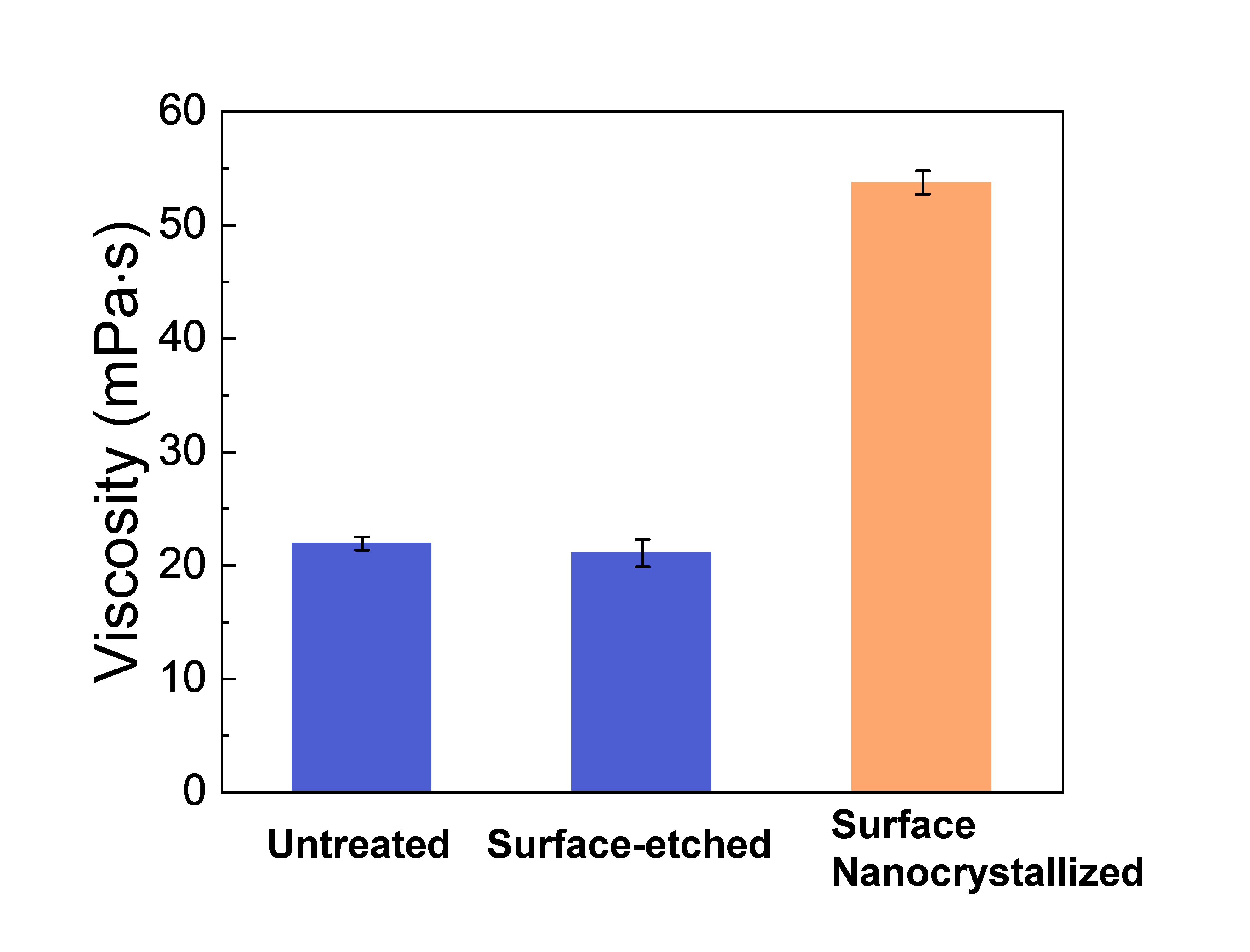


**Supplementary Figure 4. Viscosity of untreated** **wood particles, surface-etched wood particles and SNWP slurry.** The rotary viscosity of SNWP slurry can reach 53.7 mPa·s from 21.7 mPa·s for untreated wood particles.

**
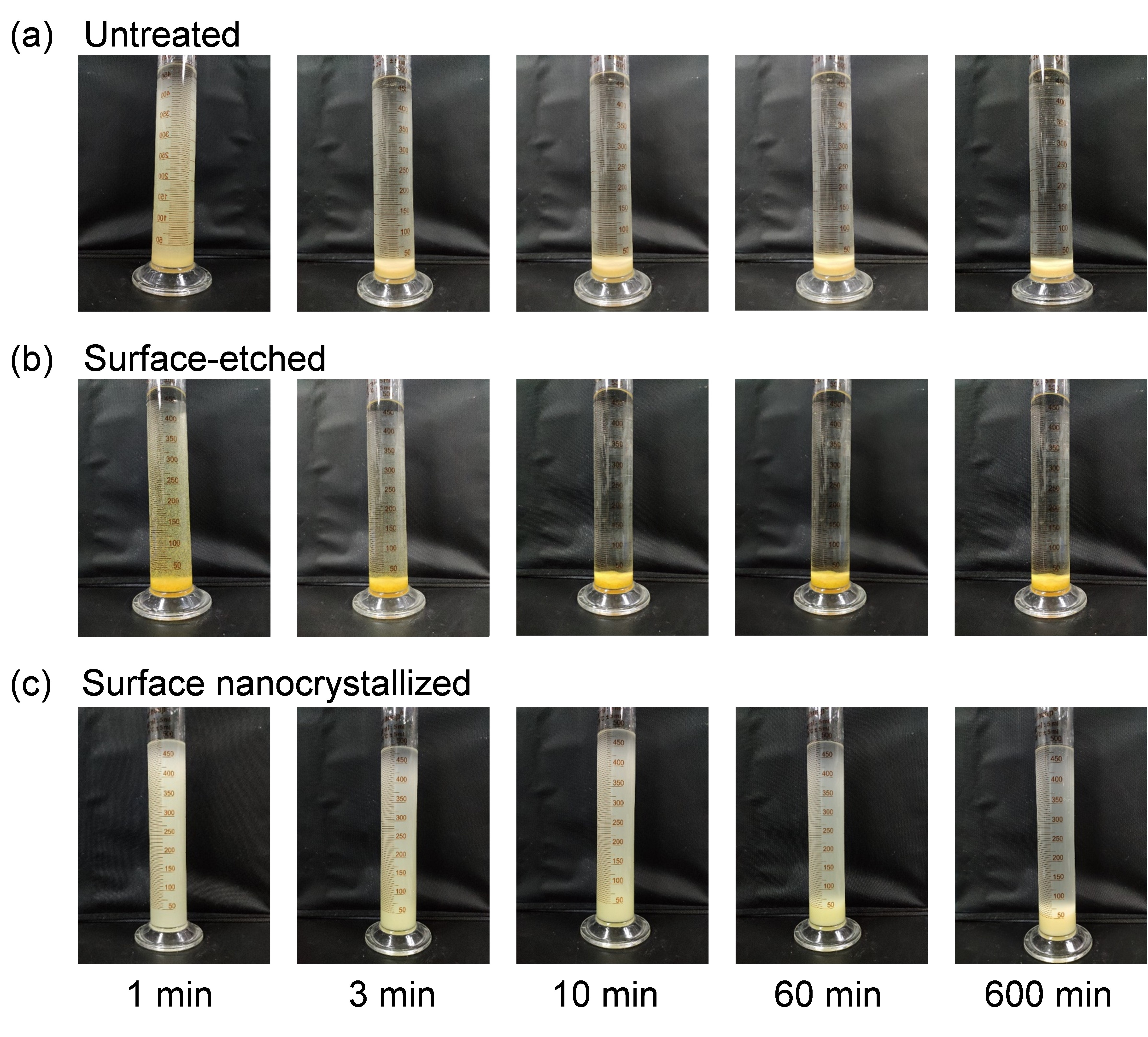
**

**Supplementary Figure 5. Photograph of settling experiment of untreated, surface-etched and surface nanocrystallized wood particles slurry.** Slurry of untreated wood particles (**a**), surface-etched wood particles (**b**) and SNWP **(c)** with solid content of 0.1 wt. % are homogenized and then poured into measuring cylinders for settling under gravity. Photograph are taken after 1, 3, 10, 60, 600 min. Untreated and surface-etched wood particles fully settle in 10 min while SNWP only partly settles after 600 min.

**
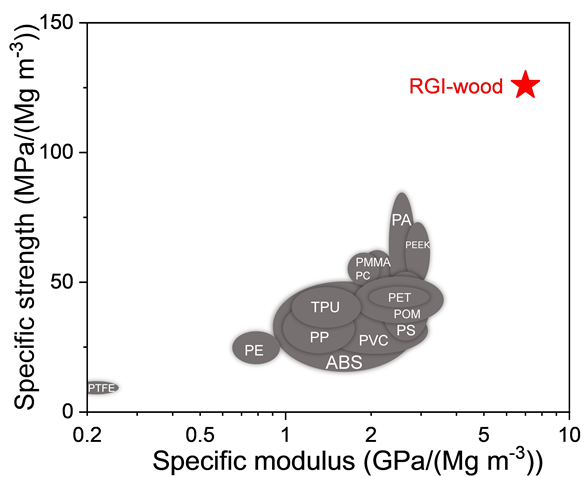
**

**Supplementary Figure 6. Ashby map of specific strength vs. specific modulus for RGI-wood compared with traditional polymers.**


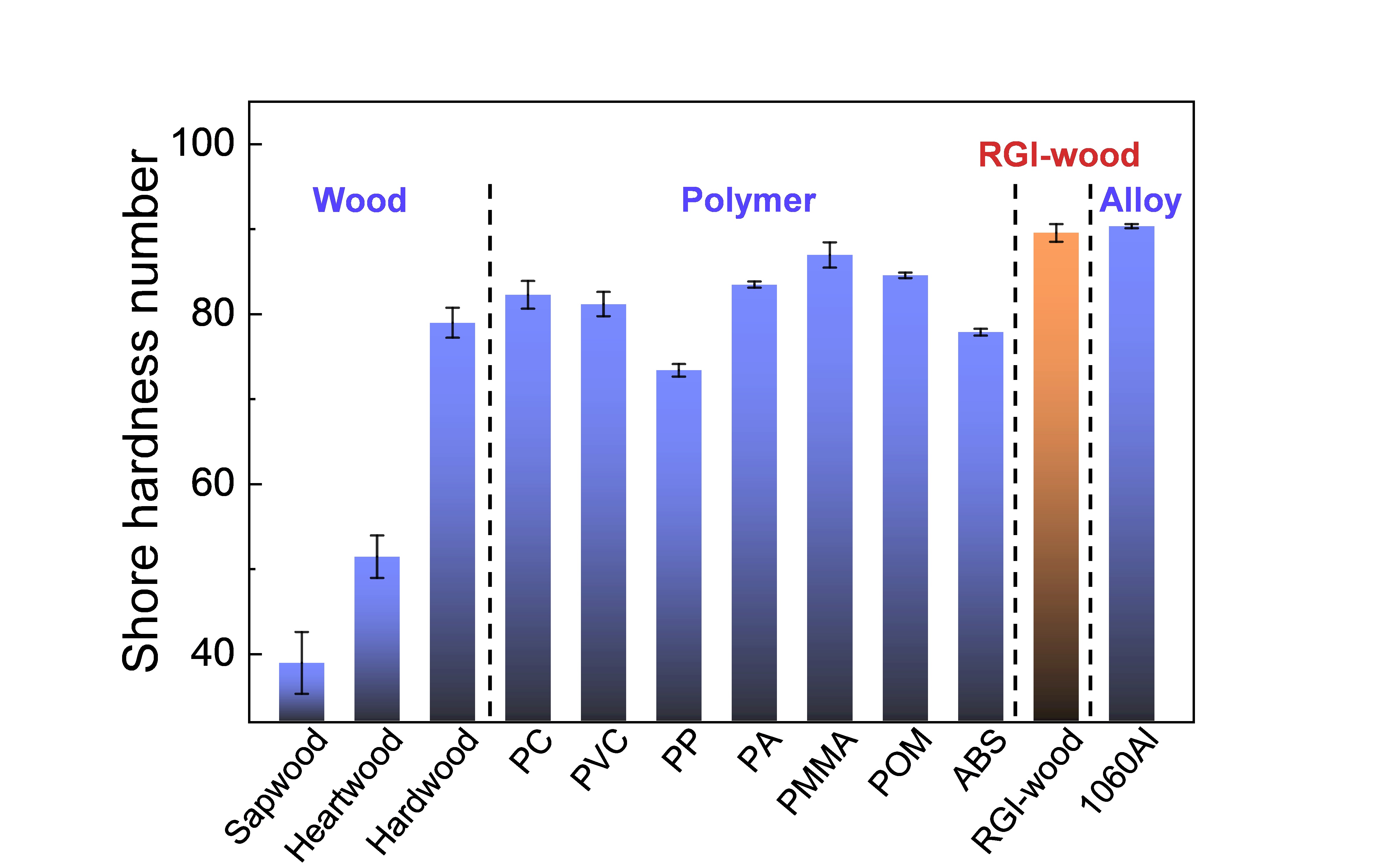


**Supplementary Figure 7. Hardness of natural wood, typical polymers, RGI-wood and 1060 Al.** RGI-wood has higher hardness than natural wood and typical polymers and its hardness is close to Al alloy.


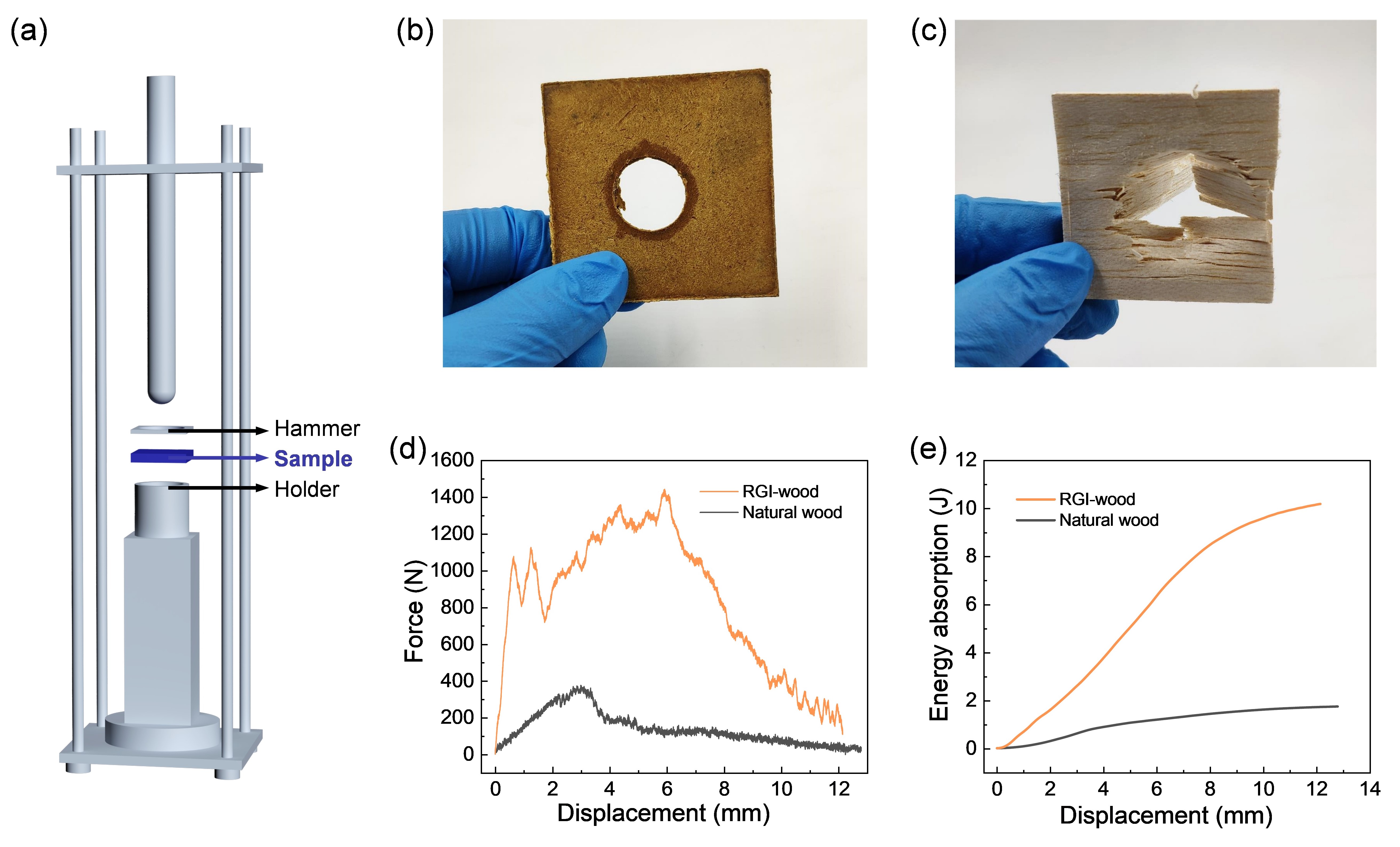


**Supplementary Figure 8. Drop hammer test of RGI-wood and natural wood.** (**a**) Schematic of the drop hammer impact tester. (**b**, **c**) Photograph of RGI-wood (b) and natural wood (c) after drop hammer impact test. (**d**) Force-displacement curves show that RGI-wood can bear larger force than natural wood. (**e**) Energy absorption-displacement curves show that RGI-wood can absorb more energy than natural wood.


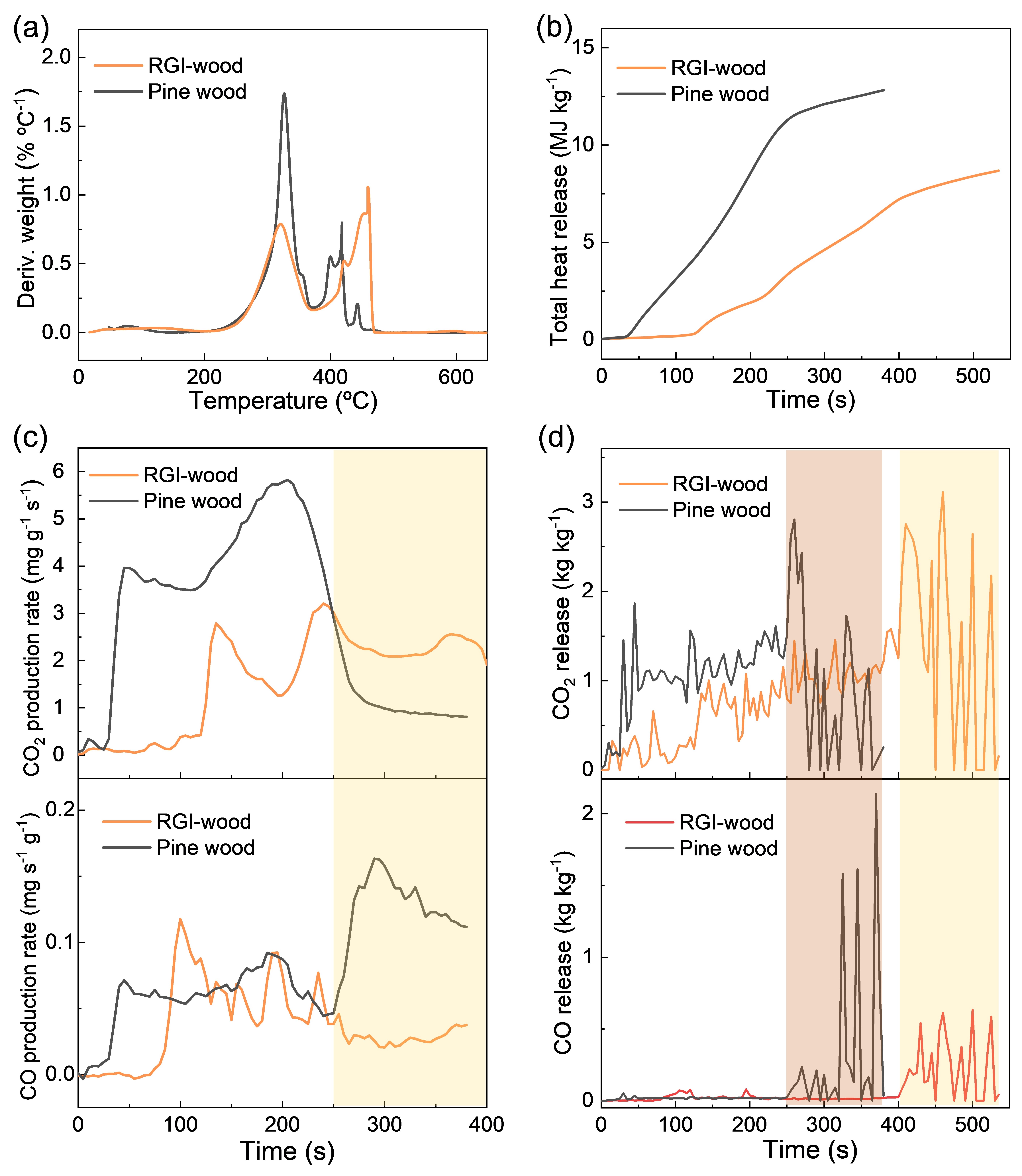


**Supplementary Figure 9. Combustion properties of RGI-wood and natural pine wood.** (**a**) DTG curves of RGI-wood and natural pine wood. (**b**) Total heat release (THR) curves of RGI-wood and natural pine wood. (**c**) CO_2_ and CO production rate curves. RGI-wood showing lower CO_2_ and CO production rate during flaming combustion and higher CO_2_ production rate and lower CO production rate during smolder (yellow area), indicating its superior fire safety to natural wood. (**d**) CO_2_ and CO release curves of RGI-wood and natural pine wood. CO_2_ and CO release means the mass of CO_2_ and CO production per kilogram sample weight loss. In the late combustion phase (red area for natural pine wood and yellow area for RGI-wood), the lost weight of RGI-wood mostly converts into CO_2_ while those of natural pine wood mostly converts into CO_._


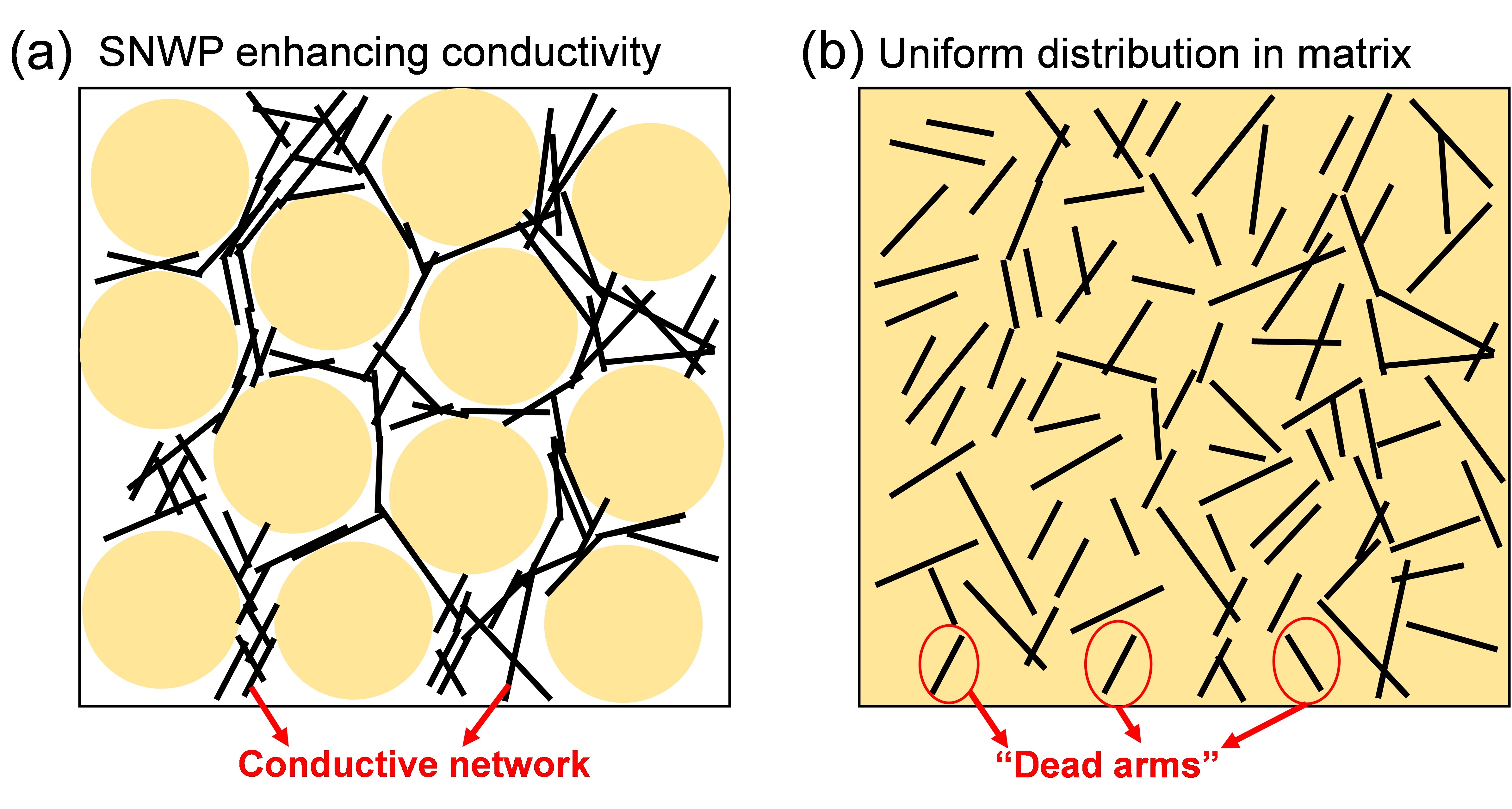


**Supplementary Figure 10. Diagrammatic sketch of SNWP enhancing conductivity and typical uniform distribution of CNTs in polymer matrix.** (**a**) Diagrammatic sketch of SNWP enhancing the contact between CNTs and reduce contact resistance. (**b**) Diagrammatic sketch of typical uniform distribution of CNTs in polymer matrix and the “dead arms”.


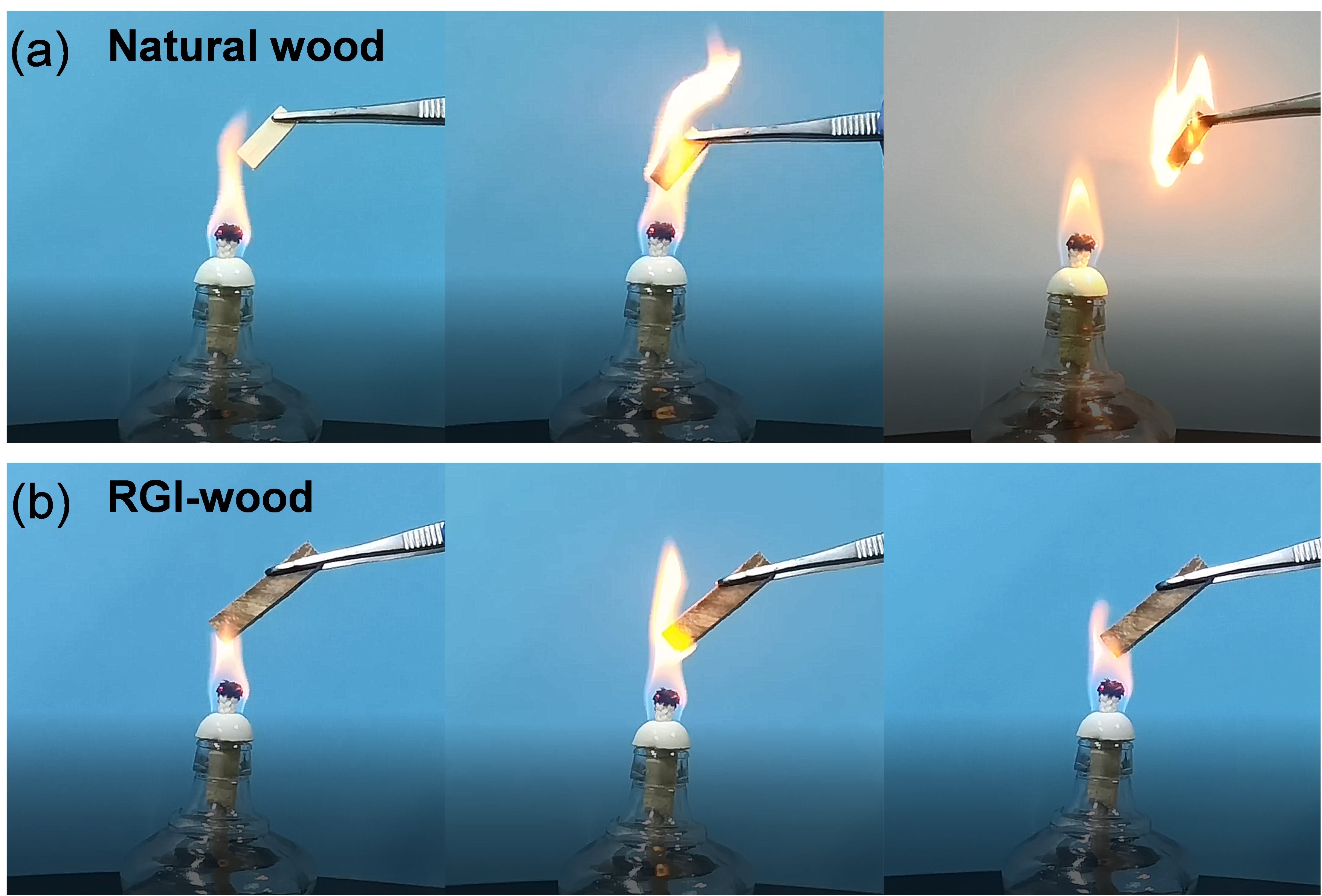


**Supplementary Figure 11. Vertical flame test.** (**a**) Natural wood. (**b**) RGI-wood.

**References**

1. R. Demir-Cakan, N. Baccile, M. Antonietti, M. M. Titirici, Carboxylate-Rich Carbonaceous Materials via One-Step Hydrothermal Carbonization of Glucose in the Presence of Acrylic Acid. *Chem. Mater.* **21**, 484-490 (2009).
